# Cholera Control and Prevention in Bangladesh: An Evaluation of the Situation and Solutions

**DOI:** 10.1093/infdis/jiy470

**Published:** 2018-08-28

**Authors:** Md Taufiqul Islam, John D Clemens, Firdausi Qadri

**Affiliations:** International Centre for Diarrhoeal Disease Research, Bangladesh

**Keywords:** Cholera, OCV, prevention, Bangladesh

Cholera, an ancient diarrheal disease, continues to be a public health threat even to this day in over 47 countries where communities are exposed to large quantities of fecal material and face problems in accessing to safe drinking water and basic hygiene.

Current global estimates reveal 2.9 million cases of cholera each year, and 95000 deaths occur from the disease annually. In Bangladesh alone, there are at least 100000 cases and approximately 4500 deaths each year [1]. One estimate suggests that more than 66 million people are at risk of cholera with an incidence rate of 1.64 per 1000 [1]. Bangladesh remains endemic for cholera with a biannual peak in certain areas of the country [2, 3]. Cholera transmission increases during both floods and droughts. Water temperature in ponds and rivers, in addition to rainfall, also has a remote association with cholera transmission [4]. Cholera affects all age groups; however, the majority of fatal cases occur in children [5, 6]. Children under 5 years of age bear a high burden, but adults are also at risk [7]. Prevention of cholera remains an important public health goal, and vaccination as well as improving access to water, sanitation, and hygiene (WaSH) are essential factors in preventing and controlling the disease [8]. Therefore, a safe, effective, and affordable vaccine is an important tool for cholera prevention and control.

An oral cholera vaccine (OCV) stockpile was established by the World Health Organization (WHO) in 2013. The number of doses released from the stockpile in response to requests from countries in Asia, Africa, and the Americas has approximately doubled every year since the stockpile’s creation, and the current supply of vaccine is currently outstripped by country demand.

An international call for ending the spread of endemic cholera is now being led by a global initiative with a plan to make at least 20 countries free of cholera by the year 2030 (#End Cholera 2030) [9]. Bangladesh is one of these countries. People living in high-risk, densely populated environments with poor access to safe water, sanitation, and education are most at risk.

Caseloads in Bangladesh are marked with peaks in seasonality, typically in the spring and autumn, in areas where bimodal seasonality is observed. People living in high-risk, densely populated environments with poor access to safe water, sanitation, and education are most susceptible. In addition, genetic factors, including ABO and Lewis blood groups as well as malnutrition in young children, can be predisposing factors. With an aim to control and prevent cholera in Bangladesh, extensive efforts have been carried out over the last decade to determine mechanisms for using OCVs to prevent the disease in Bangladesh [2, 10, 11] (Figure 1). A feasibility and effectiveness trial of OCV to assess overall protection conferred by a 2-dose regimen of Shanchol was conducted among 240000 individuals in urban Bangladesh.

**Figure 1.**
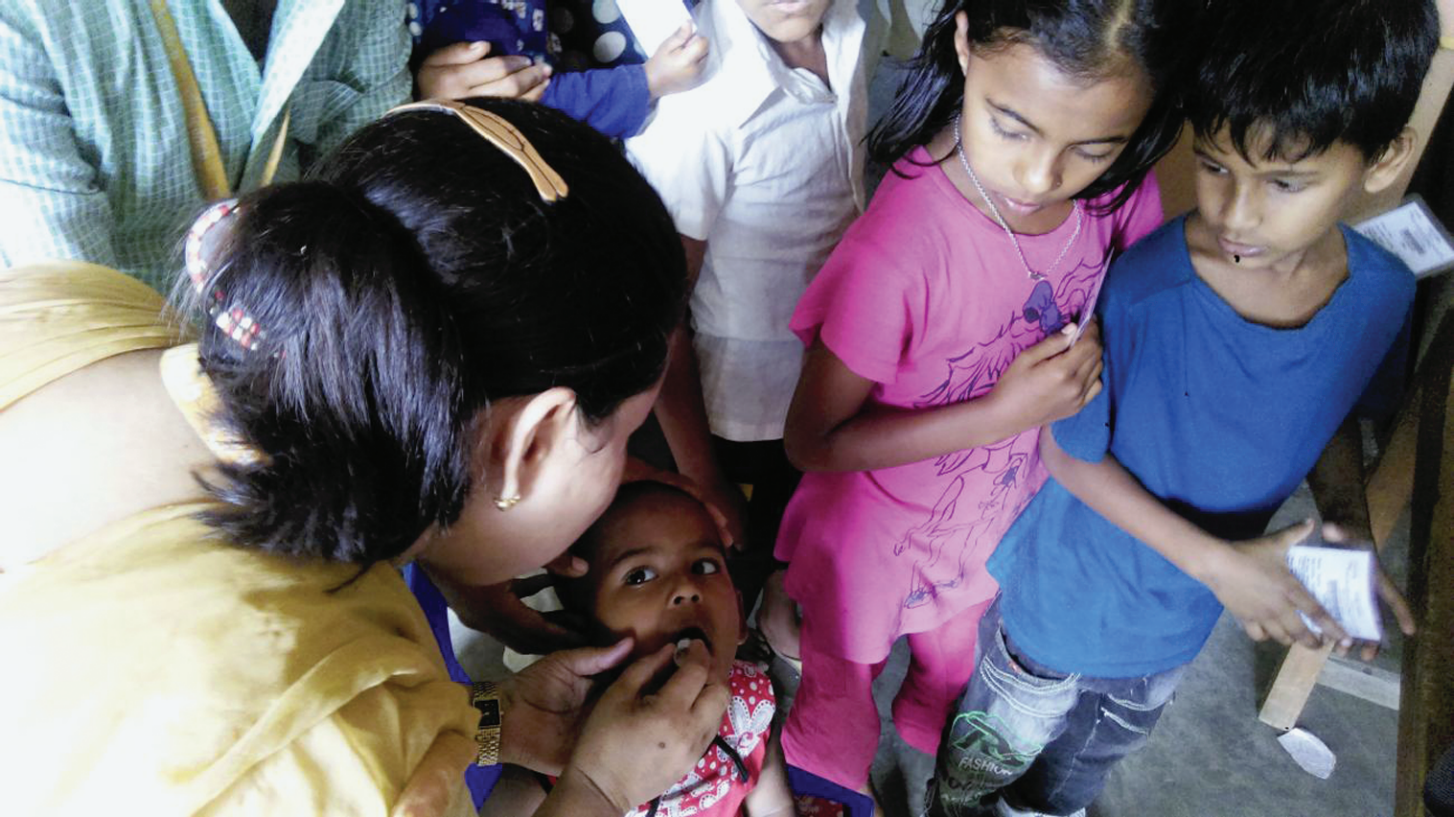
Oral cholera vaccine deployment in urban Dhaka, Bangladesh. Image reproduced with permission.

The trial showed that total protection of vaccinees for preventing hospitalization due to severe dehydrating cholera was 53% in the vaccination-only group and 58% in the vaccination and behavioral change group, which implemented hand washing and water treatment [10]. Delivery of the complete 2-dose regimen of the vaccine in the community, especially in emergency situations, can be difficult. To address whether a single dose of Shanchol is protective, a large-scale, placebo-controlled trial of Shanchol was conducted in urban Dhaka. The trial found that a single dose conferred 65% protection against hospitalizations for severe cholera during 2 years of follow-up; however, protection by a single dose was only seen in persons vaccinated at 5 years of age and older [11]. A 2-dose regimen of OCV provides less protection to children under the age of 5, and a single dose does not work in this population [12].

Futhermore, maintaining a cold chain for vaccine storage in resource-poor settings presents a major challenge to the implementation of OCV delivery. A study of Shanchol in Bangladesh found that vaccine safety and immunogenicity were not altered when the vaccine was kept at ambient temperature outside the cold chain [13].

A nationwide surveillance for cholera is being carried out in 22 sites in Bangladesh to identify hotspots of cholera and to determine areas that need immunization with OCV. Acute watery diarrhea cases attending in sites are being confirmed by microbiological culture of stool for *Vibrio cholerae*. Another initiative is that the technology of inactivated whole-cell OCVs has been transferred to a qualified producer in Bangladesh. Meanwhile, 2 inactivated whole-cell vaccines have been produced, and the later was used in Phase I/II clinical trials (NCT02742558, NCT02823899). It is important to document the effectiveness of OCV, WaSH interventions, and clinical management, and this should be a multisectoral approach for long-term control of cholera.

## CONCLUSIONS

In summary, we found that it is feasible and impactful to vaccinate the populations at risk in Bangladesh with OCV. A single dose of Shanchol can be protective in children over the age of 5 and adults, and Shanchol can be delivered in field settings without using a cold chain. However, the use of 1 dose of Shanchol, or OCVs like Shanchol, during an emergency response in a cholera-endemic setting with immunologically primed individuals may be considered in circumstances in which the supply of vaccine is limited—a likely scenario for an increasing number of cholera vaccine campaigns while the current global shortage of WHO-prequalified inactivated whole-cell OCVs persists. However, with locally produced inactivated whole-cell OCVs becoming available, we are optimistic that introduction of a complete 2-dose regimen for the population at risk in Bangladesh will be feasible in the near future. It is expected that of the 160 million people of Bangladesh, at least 66 million will need protection from cholera. However, for children under the age of 5, additional doses as well as further research on more efficacious vaccines are needed to control cholera. Meanwhile, large-scale vaccination with available vaccines can also provide indirect herd protection to children in high-risk settings.

## Notes

***Supplement sponsorship.*** This work is part of a supplement sponsored by the Bill and Melinda Gates Foundation.

***Potential conflicts of interest.*** All authors: No reported conflicts of interest. All authors have submitted the ICMJE Form for Disclosure of Potential Conflicts of Interest. Conflicts that the editors consider relevant to the content of the manuscript have been disclosed.
